# Feasibility and acceptability of telephone-based psychological support for metastatic cancer patients in Indian palliative care: An experimental study

**DOI:** 10.1017/S147895152510103X

**Published:** 2025-11-19

**Authors:** Arunima Datta, Sukriti Dutta, Prarthna Jayaseelan

**Affiliations:** 1 Clinical Psychologist, Department of Oncology, Medica Super Specialty Hospital, Unit of Manipal Hospital, Kolkata, WB, India; 2 Department of Clinical Psychology, Amity University, Noida, UP, India; 3 Department of Pain Management and Palliative Care, Medica Super Specialty Hospital, Unit of Manipal Hospital, Kolkata, WB, India

**Keywords:** palliative care, telephone-based psychological support, metastatic cancer, accessibility and acceptability, psychological well-being

## Abstract

**Objectives:**

To evaluate the accessibility and acceptability of implementing a telephone-based psychological support intervention for patients with metastatic cancer in the Indian palliative care settings.

**Materials and methods:**

The present single centered experimental study was conducted on 181 adult metastatic cancer patients who were referred to the pain and palliative medicine department by medical oncologists at a tertiary hospital, India. The patients were purposely assigned to two groups: In Group-A 90, patients received a combination of palliative and psychological support. In Group-B, 91 patients received only structured palliative care. As per the department protocol, patients in each group were followed up on days 0, 7, 14, 21, and 28. Before each session, patients completed questionnaires that are based on disease-related symptoms and psychological well-being.

**Results:**

It was found out that patients with telephone based psychological support integrated with palliative care has shown gradual improvement in physical and psychological symptoms from day 7 to day 28 when compared to the control group with *p*-value < 0.05. Additionally, 67% of patients continued their follow-up with the psychologist, indicating the accessibility and acceptability of the treatment. Furthermore, 87% of patients preferred voice calls over video calls because of the limited internet access (*N* = 72%).

**Conclusion:**

Therefore, it can be concluded that the combined approach of pain management through palliative care and continuous telephone based psychological support has contributed to their holistic well-being.

**Significance of results:**

The findings highlight that integrating telephone-based psychological support within palliative care services is both feasible and acceptable for patients with metastatic cancer in India. This approach not only improves physical and psychological outcomes but also enhances the continuity of care, especially in resource-limited settings where in-person psychological services may not always be accessible.

## Introduction

Palliative care is a structured and comprehensive approach designed to provide holistic support to individuals diagnosed with life-threatening illnesses, particularly cancer. It extends care not only to patients but also to their primary caregivers, with a focus on improving quality of life and emotional well-being. This is achieved through continuous and compassionate communication between the patient and healthcare providers (Patel et al. 2023b).

In palliative care, psychosocial health is a key area of concern, as patients often experience a decline in their mental and social well-being due to the progression of the illness. Based on previous findings, approximately 1–30% of individuals who underwent palliative care reported experiencing symptoms of depression, while around 10% reported symptoms of anxiety (Sherman and Kilby 2021). There are different causes that lead to distress among the patients and caregivers, ultimately impairing their overall quality of life (Kaur et al. 2022). Few of them are as follows. First, distress in patients undergoing palliative care often arises from the decline in physical health and bodily functions, which may result from the underlying disease or, to some extent, from treatment-related side effects (Chandra et al. 2023). Second, decision-making can be extremely challenging due to uncertainties related to the illness, the complexity of the situation, confusion, and emotional turmoil (Doval et al. 2020; Salek et al. 2023). Third, financial constraints can significantly hinder access to treatment, particularly for families with low socioeconomic status, leading to increased stress and reduced accessibility to care facilities (Kumar et al. 2018). Psychological support is crucial in such settings because it addresses a social, emotional and spiritual need of patients and their caregivers to improve their quality of life by promoting meaningful involvement in daily activities and healthcare decision-making (Kaur et al. 2021).

The COVID-19 pandemic necessitated a rapid transformation in healthcare delivery systems, positioning telemedicine as an essential modality for maintaining continuity of care while minimizing the risk of viral transmission. This shift was particularly critical in oncology and palliative care settings. Multiple studies conducted during the pandemic have demonstrated the feasibility, acceptability, and effectiveness of telemedicine in palliative care settings (Biswas et al. 2020a, 2020b). Additionally, Adhikari et al. (2021) carried out an observational study focused on audio-only teleconsultations, incorporating caregiver feedback, which revealed high satisfaction levels and supported the continued use of telephone-based services in palliative care delivery (Das et al. 2021a).

Despite the recognized importance of psychological support in palliative medicine department, there remains a significant gap in the literature regarding the impact of sustained or continuous psychological interventions on patient outcomes in this setting.

The primary objective of the present study was to assess the acceptability of continuous psychological support delivered via telephone among cancer patients receiving palliative care. Furthermore, the study evaluated dropout rates to examine the accessibility and sustainability of this mode of psychological intervention within palliative care settings.

## Materials and methods

### Study design

The present single centered experimental study conducted at a tertiary cancer hospital in eastern India after approval from the institutional review board. For the present study, we had taken two groups – experimental group received structured palliative care combined with psychological support and the other one was control group received only structured palliative care. Both groups involved data collection from the same questionnaire at 4 time points with 1 week interval.

#### Recruitment

**Figure 1.**
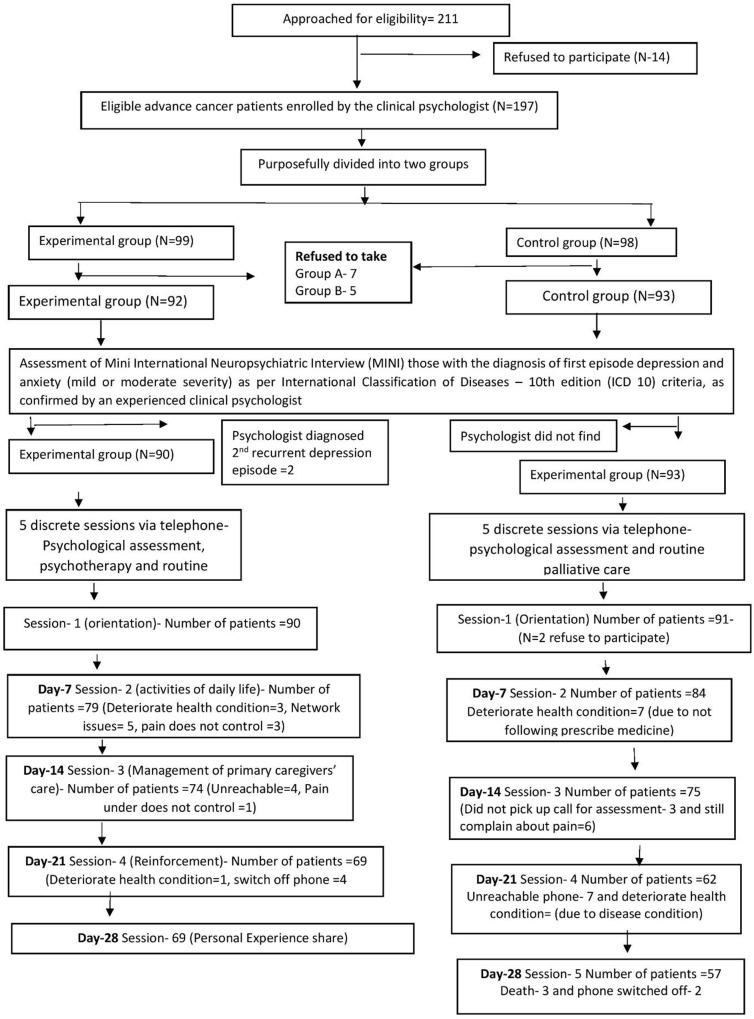
CONSORT diagram.

For the present study, out of 211 metastatic cancer patients referred to the department of pain and palliative medicine by medical oncologist between April 2024 and December 2024, only 197 patients were enrolled in the study after fulfilling eligibility criteria (Figure 1). Patients were divided into two groups: the Group A (*n* = 99) and Group B (*n* = 98) by using purposive sampling method. However, 7 participants from Group A and 5 from Group B later withdrew their consent. Consequently, these individuals were excluded from the final analysis, resulting in a sample size of Group A = 92 and Group B = 93.

Patients were screened using the Mini International Neuropsychiatric Interview (MINI). Those diagnosed with depression or anxiety, as confirmed by an experienced clinical psychologist, were included in the study. Patients with any psychiatric diagnosis other than depression or anxiety were excluded [Group A, *N* = 2].

Following initial screening, patients who met the eligibility criteria were enrolled in the study. During their first visit to the Department of Pain and Palliative Care, the study protocol was explained to each participant in a face-to-face session. Written consent was obtained, and their WhatsApp contact numbers were recorded for further communication. Participants were then requested to complete a set of baseline questionnaires, which were administered via WhatsApp by an experienced psychologist. Psychologists sent reminder messages via WhatsApp one day prior to each scheduled session. Non-responsiveness to these reminders was recorded as dropout from the study. Reasons for withdrawal or refusal to participate were also documented, providing insights into the acceptability of psychological support interventions and helping to identify key barriers to participation.

Group A

Patients were provided with integrated palliative care along with 5 sessions of psychological support via telephone. The interventions were carried out following the treatment protocol on days 0, 7, 14, 21, and 28. Before every session, the psychologist assessed symptoms and psychological well-being using validated instruments to measure the impact of the previous session.

Group B

The patients were not provided with psychological support; instead, they were solely given structured palliative service. In this service, the palliative doctor contacted them via telephone to adhere to the treatment protocol on days 0, 7, 14, 21, and 28. Additionally, the patients underwent reassessment for symptoms and psychological well-being using the identical validated tools on the corresponding days.

The recruitment eligibility criteria were the same for both groups.

### Eligibility criteria

**Inclusion criteria**

1. Diagnosed metastatic cancer patients (following radiological and clinicopathological report);
2. First visit to the palliative medicine department and had been advised to receive best supportive care;
3. No evidence of previous psychiatric illness;
4. Currently not enrolled in another psychological support study;
5. Eligible participants were adults aged greater than 18 years and less than 65 years;
6. Able to understand, read and speak the Bengali language. (Response from patients with oral cancer who were unable to verbalize was recorded with help of caregiver/ proxy.)

**Exclusion Criteria**

- Patients with a clinical diagnosis of brain metastases were excluded from the study.

### Telephone-based psychological support integrated with palliative care

Telephone-based psychological support refers to the delivery of structured mental health interventions through telecommunication, aiming to address emotional, cognitive, and behavioral concerns in patients. In the context of palliative care, this approach facilitates regular psychological counseling, promotes treatment adherence, and enhances overall well-being, particularly in resource-limited or remote settings (Kilbourn et al. 2011).

**Figure 2.**
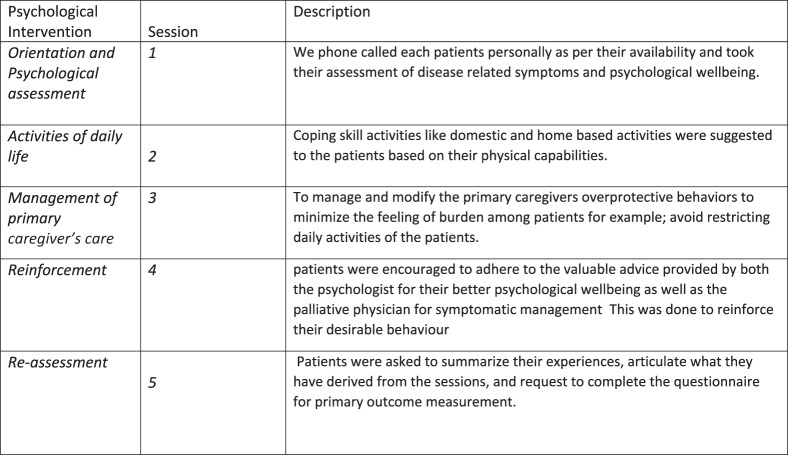
Telephone-based psychological support.

This type of psychological support was consisting of 5 sessions each lasting for 15–20 minutes. As illustrated in Fig. 2, the telephone based psychological support was constructed in five discrete sessions: (1) orientation and assessment, (2) daily life activities, (3) management of primary caregiver’s care, (4) reinforcement integration, and (5) reassessment (Fig. 2). The session content was established through its alignment with recognized psychological principles relevant to palliative care – such as cognitive-behavioral approaches, psychoeducation, and supportive counseling – and by addressing patient-identified needs such as symptom burden, caregiver interactions, and emotional resilience. The structured format allowed both flexibility and consistency, enabling tailored support while maintaining fidelity to therapeutic objectives (Yang et al. 2020).

During the first session, the primary objective was to enhance participants’ awareness of their physical and psychological symptoms. Each patient was contacted individually via telephone, based on their availability, to assess disease-related symptoms and psychological well-being. Patients were also briefly oriented to the concepts and significance of palliative care, with an emphasis on its role in ongoing symptom management and follow-up. Given the limited awareness of palliative care among the general population in West Bengal (Manna 2018), this session aimed to bridge the knowledge gap and promote engagement with supportive services.

During the second session, the patients’ recurrent physical symptoms and pain causes their self-confidence to reduce (Mir et al. 2023), so that’s why the participants were encouraged to engage in domestic and home-based activities as a coping skill to the patients after getting pain relief. The reason to suggest the patients with these coping activities was to increase their physical effectiveness. They were also able to manage their physical and psychological well-being effectively when experiencing pain.

During the third session, with the patient’s well-being, it is also important to manage and modify the primary caregivers’ overprotective behaviors or controlling behaviors (Hebert et al. 2007) following this statement the clinical psychologist request the caregivers to stop their overprotective behavior because this causes a “feeling of burden” as said by the patients. Giving space and time to palliative care patients often creates an environment to process emotions and make meaningful choices for themselves.

In the fourth session, patients were encouraged to adhere to the valuable advice provided by both the psychologist for their better psychological well-being as well as the palliative physician for symptomatic management. Most of the patients reported having improvement in their physical well-being but their psychological well-being was still a concern. So, during the fourth session, it mainly focused on reinforcing desirable behavior (domestic activities and intake of medication time) to promote emotional resilience and stability.

In the final session (Session 5), patients were asked to summarize their experiences, articulate what they have derived from the sessions telephonically and requested the patients to complete the questionnaire for primary outcome measurement (psychological and physical well-being).

### Data collection

Bengali-translated and validated versions of all tools were used to ensure accessibility and comprehension.

#### Sociodemographic details

This part includes 9 questions: present age, gender, last educational grade, relationship status (living spouse or living without spouse), living area (rural or urban), socioeconomic status (following Kuppuswamy scale), do you have family support (yes or no), do you have social support (yes or no), and do you have economical support (yes or no).

#### Clinicopathological details

Diagnosis

#### Telephone-based psychological support details

This part includes 6 questions: types of mobile (android or keypad), types of call (voice call or video call), duration of call (<15 minutes or >15–20 minutes), timings of call (9 am to 1 pm and 1 pm to 5 pm), internet access (yes or no), owner of the mobile phone (self or primary caregiver), recharge done by (self or primary caregiver), continuity of follow-up (yes or no) (Das et al. 2021b).

#### Mini International Neuropsychiatry Inventory

MINI is a brief structured diagnostic interview that was created for the DSM-IV (Diagnostic and Statistical Manual of Mental Disorders, American Psychiatric Association) by psychiatrists and physicians in the United States and Europe – 4th Edition) and ICD-10 psychiatric disorders. With an administration time of approximately 15 minutes, it is easy to administer (Datta et al. 2022).

#### Warwick-Edinburgh Mental Well-being Scale

The Warwick-Edinburgh Mental Well-being Scale (WEMWBS) questionnaire is a well-being thermometer among cancer patients. There are nine different types of questions that were pointed from none of the time = 1 to all the time = 5. Their type of questions is regarding what a patient thinks regarding their quality of life. This scale can be able to signify variation of psychological well-being with increasing raw score (Datta et al. 2016). Given the weekly structure of the intervention and the potential for rapid psychological changes in the palliative care population, WEMWBS was administered at one-week intervals, despite its standard 2-week validation interval.

#### Edmonton symptom assessment score

Edmonton symptom assessment score (ESAS) was recorded using the ESAS form as a questionnaire to rate the intensity of nine common symptoms experienced by cancer patients, including pain, tiredness, nausea, depression, anxiety, drowsiness, appetite, well-being, and shortness of breath. The total ranges from 0 to 60, with a higher score indicating higher physical symptom burden. The combined score was based on ESAS anxiety and depression. The total ranges from 0 to 20, with a higher score indicating higher emotional symptom burden (Datta et al. 2024).

### Consent form

Informed written consent was obtained from all participants and verbal consent was also recorded in audio format.

### Data analysis

SPSS version 25 was used for statistical analysis. Descriptive statistics were used to summarize patients’ demographic and clinical characteristics of all the participants. Socio-demographic, clinic-pathological characteristics WEMWBS, ESAS scores were categorized according to two participated groups. Chi-square was applied to observe comparability according to two cancer groups following demographic variables. The overall mean values of each scale at baseline/day 0, day 7, 14, 21, and day 28 of telephone based psychological support course were calculated for both groups. Repeated measure ANOVA was used to define significance of telephone based psychological support among metastatic cancer patients through psychological assessment score before, during and after participation. After the intervention, patients’ physical symptoms and psychological well-being were compared between the experimental and standard care groups. These variables were selected to evaluate the clinical and psychosocial impact of the intervention.

### Ethics approval

The study was conducted after approval from the institutional review board followed by **The Indian Council of Medical Research** (ICMR), CREC/2020/April/1 (ii).

### Results

#### Demographic information

Table 1 depicts the baseline demographic and clinical characteristics of the study participants. According to the group division.

**Table 1.** Prevalence of demographic and clinicopathological details among participants

Demographic details	Group A (*N* = 90)	Group B (*N* = 91)	*p*-Value
*Age*	45.23 ± 2.05	51.07 ± 2.19	0.002*
*Gender*			
Male	69%	81%	1.13
Female	31%	19%	
*Residence*			
Rural	39%	54%	0.097
Urban	61%	46%	
Relationship status			
Living with spouse	74%	65%	1.14
Living without spouse	26%	35%	
Education			
Illiterate	9%	2.5%	0.003*
Primary Education	17%	20.1%	
Secondary Education	31%	26.1%	
Graduate	26%	33.2%	
Master	12%	9%	
Socioeconomic status			
Lower middle class	13%	12%	1.31
Middle class	59%	61%	
Upper middle class	28%	27%	
Family support			
Yes	38%	41%	0.79
No	62%	59%	
Social support			
Yes	23%	22.5%	0.87
No	77%	77.5%	
Economic support			
Yes	29%	27%	
No	71%	73%	
Clinicopathological			
Diagnosis			
Oral cavity	20%	16.5%	1.01
Thorax	22%	17.6%	
Gynecological	3.5%	10.2%	
Gastrointestinal	15.5%	17.1%	
Brain	12%	9.1%	
Bone and soft tissue	5%	4.9%	
Blood	19%	18.1%	
Genitourinary	3%	6.5%	

In Group A, the mean age of participants was 45.23 ± 2.05 years. Of the participants, 69% were male, 61% were residing in urban area, 74% were living with their spouse, and 31% had received education up to secondary level. Distribution of participants according to the cancer site: oral cavity (20%), thorax (22%), and gastrointestinal tract (15.5%). The family support is present in 38% of patients, whereas the social support is present in 23% of patients.

In Group B, the mean age of participants was 51.07 ± 2.19 years. Of the participants, 81% were male, 46% resided in urban areas, 65% were living with their spouse, and 26% had received secondary education.

Distribution of participants according to the cancer site: oral cavity (16.5%), thorax (17.6%), gastrointestinal tract (17.1%), and blood (18.1%).

All groups were comparable in terms of sociodemographic (except age and education), clinicopathological, and psychological variables. The family support is present in 41% of patients, whereas the social support is present in 22.5% of patients.

All groups were comparable in terms of sociodemographic (except age and education), clinicopathological, and psychological variables.

#### Telephone-based psychological support characteristics

Table 2 represents the characteristics of telephone-based psychological support among participants receiving palliative care. The results are found that 87% of people prefer voice call over video call, only 28% of patients have access to internet, and 67% of patients continued their follow-up in the whole study period.

**Table 2.** Telephone-based psychological support characteristics among Group A

Type of mobile phone	Percentage
Android	32%
Keypad	68%
Types of call	
Voice	87%
Video	23%
Duration of calls	
<15 minutes	21%
>15–20 minutes	79%
Timing of calls	
9 am–1 pm	83%
1 pm–5 pm	17%
Internet access	
Yes	28%
No	72%
Owner of the mobile phone	
Self	76%
Primary caregiver	34%
Recharge done by	
Self	21%
Primary caregiver	79%
Continuity of follow-up	
Yes	67%
No	33%

#### Outcomes variables

Mean total scores for physical and psychological symptoms for both the study groups were presented at baseline and at days 7, 14, 21, and 28. We observed a statistically significant (*p* < 0.05) decrease in the total mean scores across all study outcomes, including ESAS (45.2 → 32.19 → 28.41 → 24.39 → 20.4) and psychological well-being (14.23 → 15.3 → 14.3 → 18.6 → 19.1) in Group A at all-time points when compared to Group B: ESAS (45.67 vs 35.1 vs 34.89 vs 34.7 vs 33.54) and psychological well-being (14.11 vs 14.9 vs 11.56 vs 12.8 vs 13.3) (Table 3).

**Table 3.** Prevalence of psychological variables over period of psychological intervention

	Mean ± SD					
	ESAS			Psychological well-being		
Day	Group A	Group B	*p*-Value	Group A	Group B	*p*-Value
Day 0	45.2 ± 1.15	45.67 ± 2.23	1.89	14.23 ± 2.01	14.11 ± 1.75	1.11
Day 7	32.19 ± 1.02	35.1 ± 1.1	0.000*	15.3 ± 1.1	14.9 ± 2.2	0.034*
Day 14	28.41 ± 1.4	34.89 ± 1.63	0.003*	14.3 ± 2.14	12.56 ± 1.05	0.021*
Day 21	24.39 ± 2.1	34.7 ± 2.13	0.000*	18.6 ± 1.08	16.8 ± 2.3	0.004*
Day 28	20.4 ± 1.05	33.54 ± 2.19	0.000*	19.1 ± 2.24	15.3 ± 1.1	0.012*

* *p* < 0.05.

There was a statistically significant interaction in telephone-based psychological support between two groups: between treatment – *F* = 4.76, *df* = 1, and *p* = < 0.005; between follow-up – *F* = 3.07, *df* = 1.67, *p* = < 0.005 (Table 4).

**Table 4.** Repeated measure one way ANOVA

Source of variation	SS	*df*	MS	*F*	*p*-Value
**Between treatment**					
Group A	60.05	1	60.04	5.35	0.000
l Group B	60.31	2	81.45	2.01	0.000
Group A × Group B	4.16	2	1.23	4.76	0.001
Error	84.88	41	0.22		
**Within follow-up**					
Follow-up	6.31	0.78	5.77	8.91	0.000
Follow-up × experimental group	3.92	0.78	2.18	3.3	0.047
Follow-up × control group	0.65	1.59	4.29	6.48	0.000
Follow-up × Group A × Group B	1.79	1.67	2.45	3.07	0.011
Error	2.31	5.47	0.66	0.6	

## Discussion

Our study explored the acceptability and effectiveness of telephone-based psychological support when integrated into palliative care settings for cancer patients. The findings suggest that this combined approach had a positive impact on patients’ physical and psychological well-being. Three primary areas contributed to the intervention’s acceptability: continuous follow-up, symptom management, and improved psychological well-being.

Patients who received regular calls from a psychologist (Group A) demonstrated better adherence to follow-up schedules compared to those in the control group (Group B), who received only standard palliative care. Structured, proactive telephone-based psychological support leads to higher adherence and reduced dropout, particularly in oncology and palliative care settings. This scientific backing strengthens the credibility of our results showing improved follow-up and acceptability in Group A (Figure 1). The literature consistently shows that structured telephone outreach improves patient retention, treatment adherence, and engagement in cancer and palliative care settings (Miller et al. 2013; Wenzel et al. 2015).

In terms of outcomes, there was a gradual improvement in both physical and psychological symptoms from pre-treatment to post-treatment, and further from post-treatment to follow-up (Table 3). Physical symptoms such as appetite loss, nausea, pain, and sleep disturbances were alleviated through appropriate palliative medications. Psychological well-being, as measured by the Edmonton Symptom Assessment Scale (ESAS), also improved notably in Group A. These improvements were not as evident in the control group. The inclusion of telephone-based psychological support thus contributed meaningfully to holistic patient care. These results align with previous research, such as the work of psychological support helps address emotional burdens such as depression and anxiety, which are often expressed through somatic symptoms (Volpato et al. 2023). Our findings reinforce this view, as patients in Group A demonstrated lower ESAS scores and greater emotional stability (Table 4).

Although previous studies have highlighted limitations to providing psychological support in oncology settings – including issues related to access, communication, patient awareness, and stigma (Weis 2015) – our findings suggest that a structured and proactive approach can help overcome these barriers. The regular, scheduled phone calls provided a consistent point of contact, which appeared to reduce stigma and encourage patient participation.

However, accessibility remains a key limitation. In our study, 59% of participants came from middle-class socioeconomic backgrounds, where affordability of mobile recharges or smartphones with internet access posed challenges. These financial constraints led to missed follow-up calls and, in some cases, a complete loss of contact. While telemedicine has been touted as a feasible solution for broader healthcare delivery in India due to the widespread use of mobile technology (Patel et al. 2023a), our findings highlight that mobile ownership and internet access are still inconsistent, especially in lower-resource settings.

Despite these limitations, our study supports the feasibility and acceptability of telephone-based psychological support as a **complementary intervention** in palliative care. It enhances both physical symptom control and emotional resilience, and, when implemented systematically, it has the potential to overcome barriers that have traditionally limited psychological care in oncology.

### Study strength

- Contribution to limited literature: The study addresses a significant gap in the Indian palliative care context by evaluating a non-pharmacological, scalable mental health intervention, contributing to the growing body of evidence supporting integrated psychosocial care.
- Real-world applicability: Conducted in a real-life clinical setting, the study offers practical insights into how telephone-based psychological interventions can be implemented in routine palliative care, especially where in-person consultations are limited.

### Study limitations

- Single-center study: The study was conducted at a single tertiary care institution, which may affect the generalizability of the findings to other settings, particularly rural or resource-limited environments.
- Absence of randomization: The absence of a randomized control design limits causal inferences regarding the effectiveness of the telephone-based psychological intervention.
- Short follow-up period: The follow-up duration was relatively short (28 days), which may not fully capture the long-term psychological impact of the intervention or its sustainability.

## Conclusion

Telephone-based psychological support should be recognized as an essential component of care in pain and palliative care settings. It has the potential to be integrated into routine practice to monitor and improve patients’ psychological well-being. Based on our study findings, we conclude that when palliative physicians make timely referrals to psychologists, and follow-up is maintained through regular telephone consultations, there is noticeable improvement in both physical and psychological health outcomes. In alignment with the principles of palliative care, we propose the implementation of a structured psychological intervention in future initiatives, with the aim of enhancing and maintaining the psychological well-being of caregivers.

Even for patients with limited mobility, regular telephone-based communication ensures continuity of care and adherence to follow-up. Moreover, initiatives such as the Pallium India free telehealth helpline, which offers psychological support to patients under palliative care, serve as a valuable model. Incorporating similar services into urban, corporate hospital settings could significantly improve the management of both physical and emotional symptoms, especially for patients with restricted access to in-person consultations.
